# Supplementary material for: A Newly Validated HPLC-DAD Method for the Determination of Ricinoleic Acid (RA) in PLGA Nanocapsules
Source: Pharmaceuticals (Basel). 2024 Sep 17;17(9):1220. doi: 10.3390/ph17091220 (PMC11435140; doi:10.3390/ph17091220)
Supplement: Supplementary file 1 [file pharmaceuticals-17-01220-s001.zip › pharmaceuticals-3125083-supplementary.pdf]

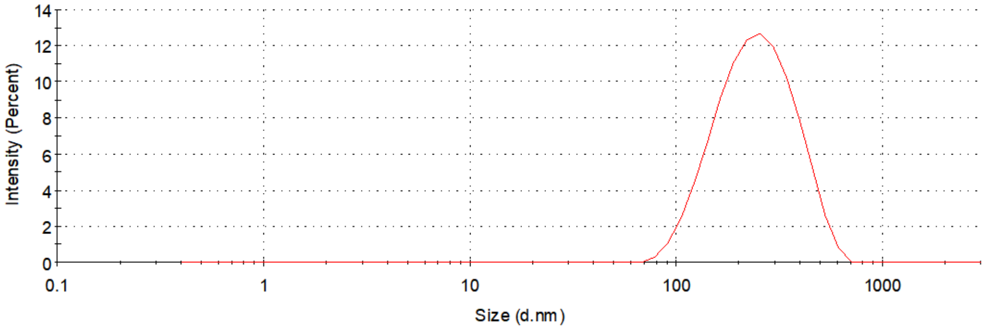
Intensity (Percent)
14
12
10
8
6
4
2
0
0.1
1
10
100
1000
Size (d.nm)

(a)

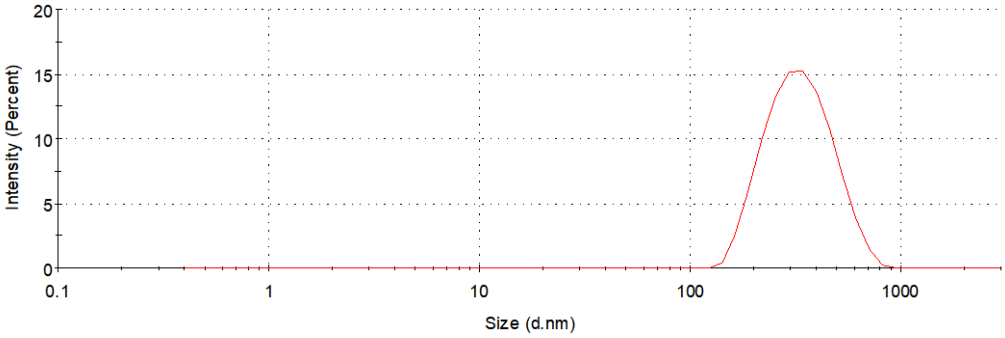
Intensity (Percent)
20
15
10
5
0
0.1
1
10
100
1000
Size (d.nm)

(b)

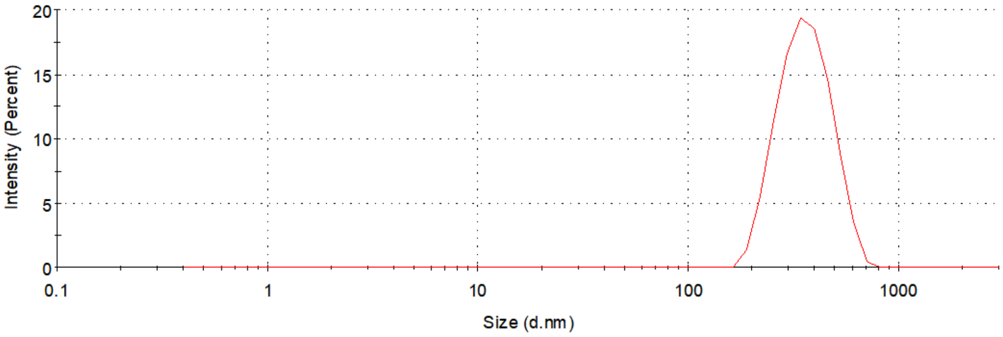
Intensity (Percent)
20
15
10
5
0
0.1
1
10
100
1000
Size (d.nm)

(c)

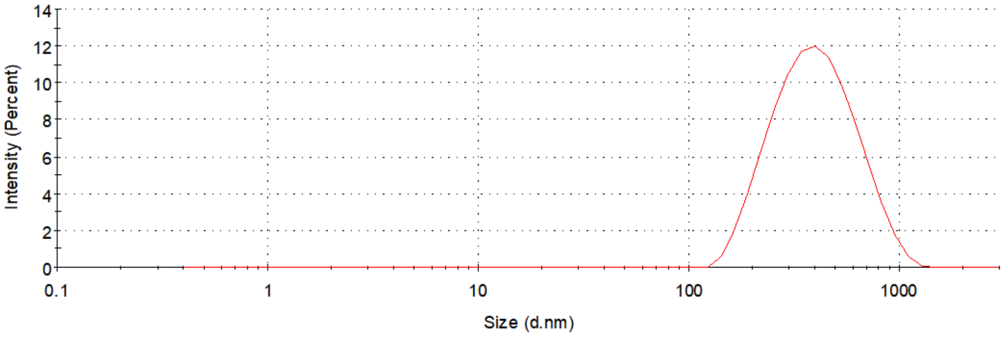
Intensity (Percent)
14
12
10
8
6
4
2
0
0.1
1
10
100
1000
Size (d.nm)

(d)

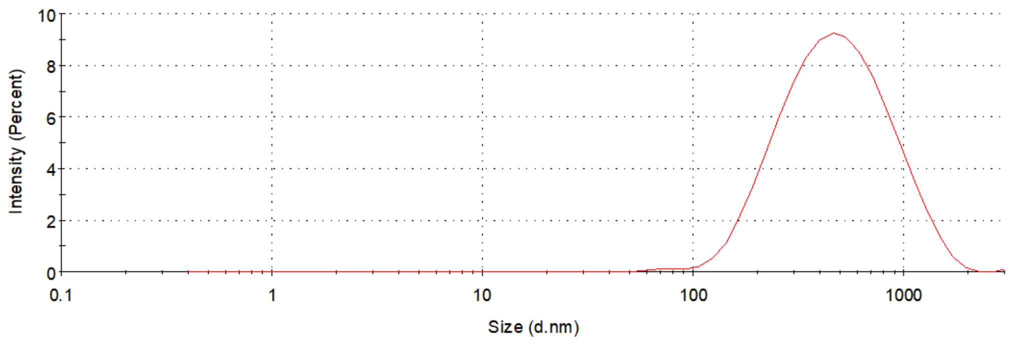

(e)

Figure S1. Particle size distributions of (a) blank optimized nanocapsules (Blank NP); (b) NCRA1; (c) NCRA2; (d) NCRA3 and (e) NCRA4.

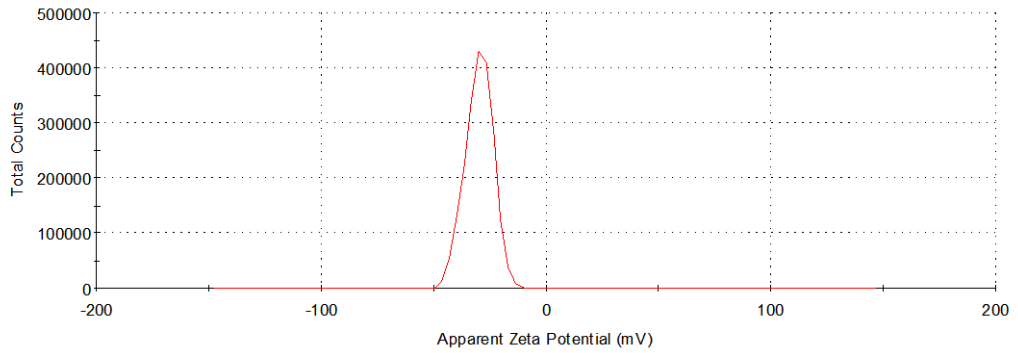

(a)

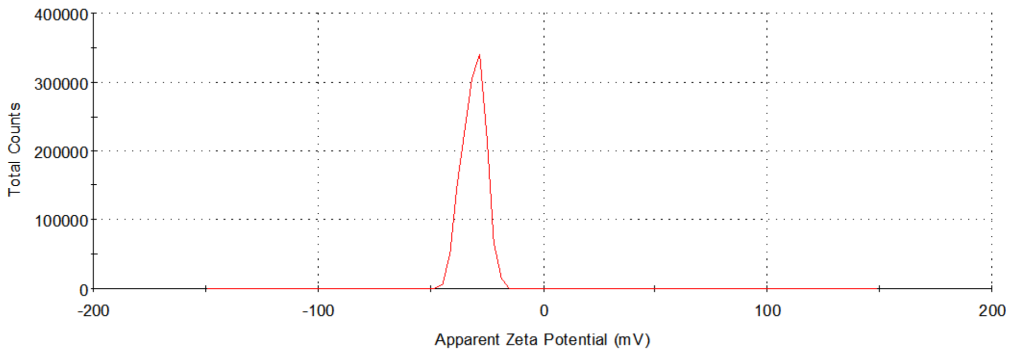

(b)

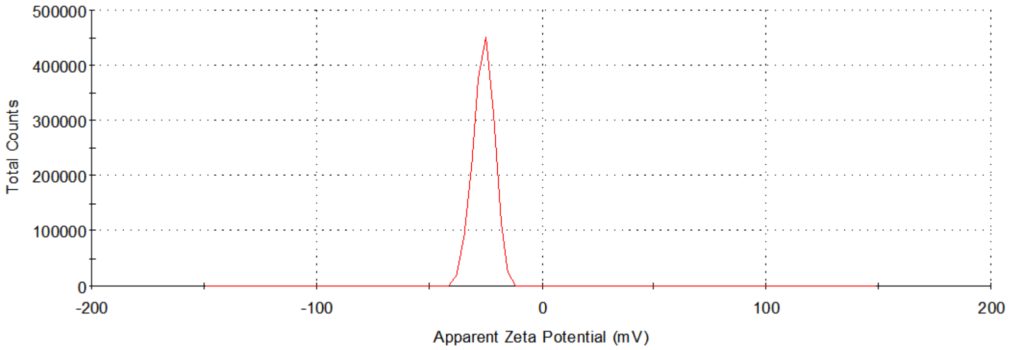

(c)

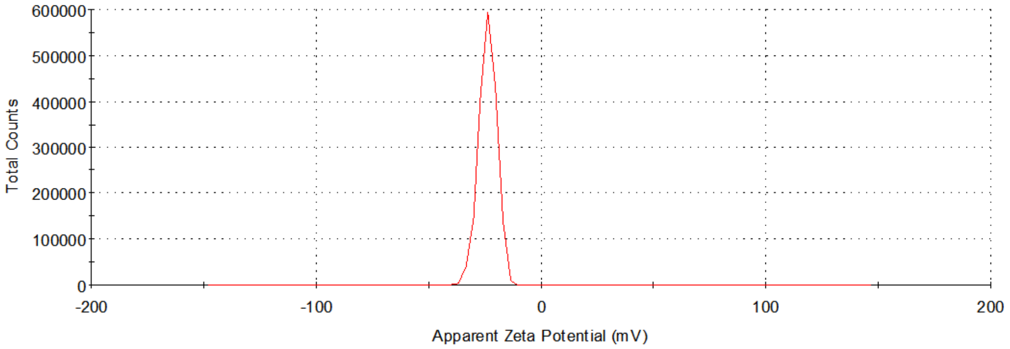

(d)

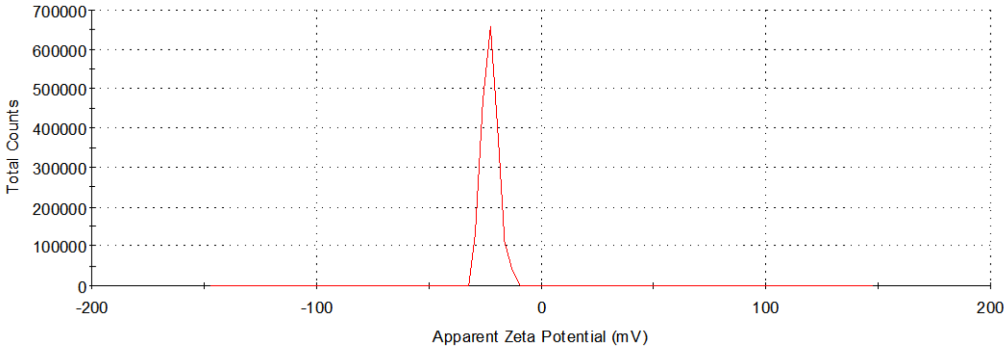

(e)

Figure S2. Zeta potential (ZP) distributions of (a) blank optimized nanocapsules (Blank NP); (b) NCRA1; (c) NCRA2; (d) NCRA3 and (e) NCRA4.

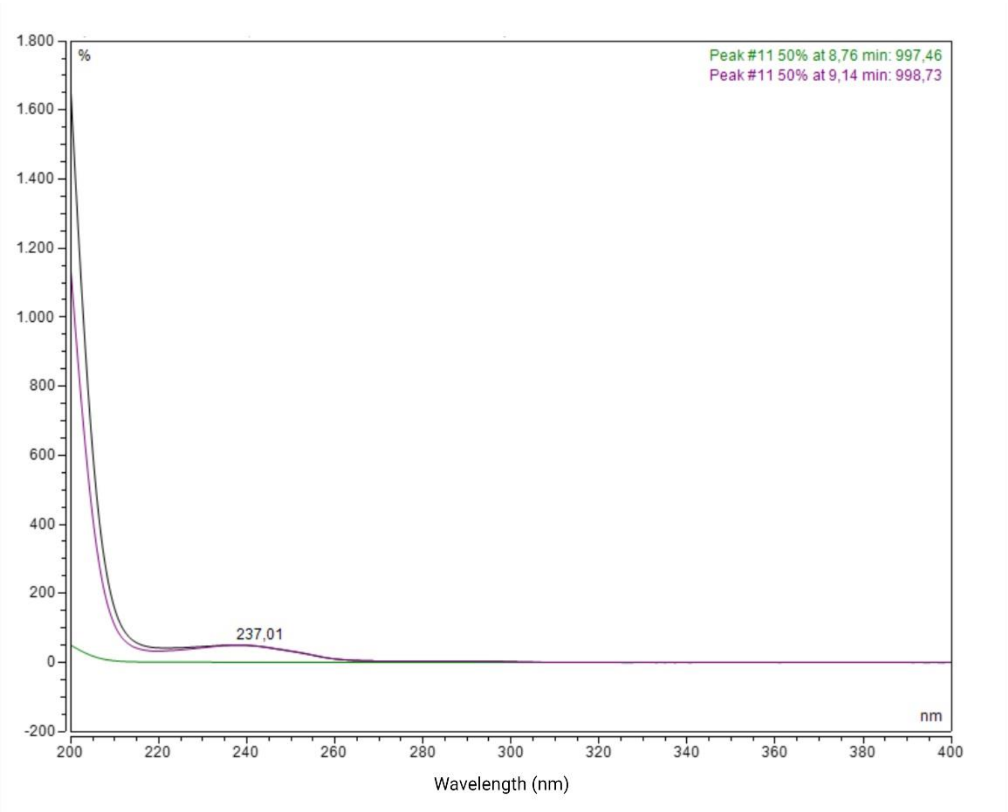

Figure S3. Maximum absorption peaks of ricinoleic acid.
